# Health professionals' knowledge and attitude towards patient confidentiality and associated factors in a resource-limited setting: a cross-sectional study

**DOI:** 10.1186/s12910-022-00765-0

**Published:** 2022-03-14

**Authors:** Masresha Derese Tegegne, Mequannent Sharew Melaku, Aynadis Worku Shimie, Degefaw Denekew Hunegnaw, Meseret Gashaw Legese, Tewabe Ambaye Ejigu, Nebyu Demeke Mengestie, Wondewossen Zemene, Tirualem Zeleke, Ashenafi Fentahun Chanie

**Affiliations:** 1 Department of Health Informatics, Institute of Public Health, College of Medicine and Health Sciences, University of Gondar, Gondar, Ethiopia; 2 Department of Health Informatics, College of Medicine and Health Sciences, Debre Markos University, Debre Markos, Ethiopia; 3 Department of Health Information Technology, Debre Berhan Health Science College, Debre Berhan, Ethiopia; 4 Health Management Information System Unit, Mekaneselam Hospital, Mekaneselam, Ethiopia; 5 Department of Health Information Technology, Teda Health Science College, Teda, Ethiopia

**Keywords:** Health professional, Knowledge, Attitude, Confidentiality, Ethiopia

## Abstract

**Background:**

Respecting patients’ confidentiality is an ethical and legal responsibility for health professionals and the cornerstone of care excellence. This study aims to assess health professionals’ knowledge, attitudes, and associated factors towards patients’ confidentiality in a resource-limited setting.

**Methods:**

Institutional based cross-sectional study was conducted among 423 health professionals. Stratified sampling methods were used to select the participants, and a structured self-administer questionnaire was used for data collection. The data was entered using Epi-data version 4.6 and analyzed using SPSS, version 25. Bi-variable and multivariable binary logistic regression analyses were used to measure the association between the dependent and independent variables. Odds ratio with 95% confidence intervals and *P* value was calculated to determine the strength of association and to evaluate statistical significance.

**Result:**

Out of 410 participants, about 59.8% with [95% CI (54.8–68.8%)] and 49.5% with [95% CI (44.5–54.5%)] had good knowledge and favorable attitude towards patents confidentiality respectively. Being male (AOR = 1.63, 95% CI [1.03–2.59]), taking training on medical ethics (AOR = 1.73, 95% CI = [1.11–2.70]), facing ethical dilemmas (AOR = 3.07, 95% CI [1.07–8.79]) were significantly associated factors for health professional knowledge towards patients’ confidentiality. Likewise, taking training on medical ethics (AOR = 2.30, 95% CI [1.42–3.72]), having direct contact with the patients (AOR = 3.06, 95% CI [1.12–8.34]), visiting more patient (AOR = 4.38, 95% CI [2.46–7.80]), and facing ethical dilemma (AOR = 3.56, 95% CI [1.23–10.26]) were significant factors associated with attitude of health professionals towards patient confidentiality.

**Conclusion:**

The findings of this study revealed that health professionals have a limited attitude towards patient confidentiality but have relatively good knowledge. Providing a continuing medical ethics training package for health workers before joining the hospital and in between the working time could be recommended to enhance health professionals’ knowledge and attitude towards patient confidentiality.

**Supplementary Information:**

The online version contains supplementary material available at 10.1186/s12910-022-00765-0.

## Introduction

Confidentiality refers to the restriction of access to personal information from unauthorized persons and processes at authorized times and in an authorized manner [1, 2]. When we say patients have the right to confidentiality, it refers to keeping privileged communication secret and cannot be disclosed without the patient’s authorization [3, 4].

Health professionals have a legal obligation to handle patients' information privately and securely [5]. As a result, patients and professionals develop trust and a positive relationship. If such highly sensitive data is improperly disclosed, it could threaten patients' safety [6]. Hence confidentiality needs to be respected to protect patients’ well-being and maintain society’s trust in the physician–patient relationship. The issue of confidentiality has been recognized as a global concern. As a result, several internationally agreed on principles and guidelines for maintaining the sanctity of patients’ private lives during treatment. This law, known as Data Protection Act, was enacted in 1998 and was last revised in 2018 [7, 8]. The Data Protection Act was created to provide protection and set guidelines for handling personal data [7]. There is no comprehensive data protection law in Ethiopia that covers health data protection [9]. Ethiopia's only confidentiality-oriented policy is the healthcare administration law, which requires health practitioners to maintain confidentiality. This law mandates health providers to keep patients' health information confidential [10]. Furthermore, only a few research have looked into health professionals' awareness of ethical rules and data security and sharing laws in Ethiopia [9].

Confidentiality is the basis of the legal elements of health records and an ethical cornerstone of excellent care [11]. More importantly, the quality of information shared with healthcare experts is determined by their capacity to keep it private. Otherwise, the patient may withhold important information, lowering the quality of care offered.

Although information sharing is essential in an interdisciplinary health team, each professional should limit information disclosure to an unauthorized health professional to plan and carry out procedures in the patient's best interests [12]. The exchange of patient medical records and data with an unauthorized person continues to be a common occurrence in a variety of clinical settings [5]. Breaches of confidentiality in clinical practice due to negligence, indiscretion, or sometimes even maliciously jeopardize a duty inherent in the physician–patient relationship [8]. Breaches of confidentiality and sharing data with unauthorized parties may have the potential to harm the patients’ health [13]. Health care quality declines due to a loss of confidence in the professional-patient relationship [14]. Patients become hesitant to seek care and attend follow-up appointments due to their mistrust of health providers [7, 8].

Until recently, the standard curricula of Ethiopia's recent medical schools did not include a medical ethics course. Nevertheless, following proposals from the Ethiopian Medical Association and curriculum review committees, the medical ethics course was first established at Addis Ababa University's Faculty of Medicine in 2004 [15]. Despite the existence of a medical ethics course, patients' concern about maintaining their confidentiality has grown, and reports of unethical behavior by health professionals on patient confidentiality are familiar [15].

There are so many problems regarding patient medical record confidentiality and data sharing [16]. The loss of patient medical records due to handling by unauthorized staff without consent and transporting to another department is a big issue in Ethiopia. That can affect patients’ quality of care by consuming time, harming patient satisfaction, causing improper diagnosis, and making it difficult to get the previous history.

The significance of this research is that it addresses the rapidly growing trend of patient data sharing and confidentiality among health practitioners in developing countries taking Ethiopia as an example. There is limited evidence regarding health professional knowledge and attitude related to patients’ confidentiality in resources limited settings. Therefore, this study will fill evidence gaps on health professional knowledge, attitude, and associated factors related to patient confidentiality in Ethiopia. This study will provide policymakers with up-to-date information on health professionals' knowledge and attitude towards patient confidentiality. Aside from that, the outcomes of this study may aid legislators in developing plans to improve health professionals' knowledge and attitude toward patient confidentiality.

## Method

### Study design and setting

An Institutional based cross-sectional study was conducted among health professionals from August–September 2021. Gondar is a historical town situated in the northwestern part of Ethiopia, 772 km far from the capital Addis Ababa and 168 km from Bahir Dar [17]. The University of Gondar specialized hospital is one of the largest teaching hospitals in the Amhara region providing tertiary level care for more than seven million people in the northwest part of the country coming from Amhara, Tigray, and Benishangul Gumuz regions [18]. It has 960 health professionals distributed over 30 services units responsible for delivering healthcare services to an average of 800 patients per day.

### Study subjects and eligibility criteria

All healthcare professionals working in the University of Gondar specialized hospital and those available during the study period were the sources and study population. The study excluded health professionals with less than six months of experience, those who had not been found in the hospital for various reasons, and those on yearly leave during the data collection period.

### Sample size determination and sampling procedure

The sample size was calculated using the single population proportion formula, n = Z(α/2)^2^ pq/d^2^ [19]. We assumed: n = the required sample size, Z = the value of standard normal distribution corresponding to α/2, 1.96, p = proportion of health professionals who had good knowledge and attitude towards confidentiality, q = proportion of health professionals who had unfavorable knowledge and attitude towards confidentiality, and d = precision assumed as 0.05. To our knowledge, no study has been conducted in Ethiopia to determine the knowledge and attitude of health professionals towards patient confidentiality. Therefore, we assumed p (proportion of health professionals who had good knowledge and attitude towards confidentiality) to be 0.5. Hence, the required sample size was calculated to be 384. After adding a 10% non-response rate, 423 health care professionals were enrolled in the study.

**Fig. 1:**
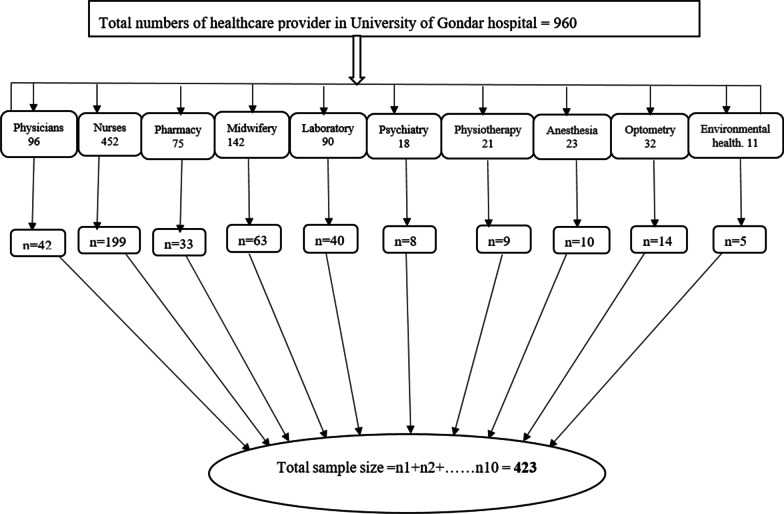
Sampling procedure and sample allocation in University of Gondar hospital

Stratified with a simple random sampling method was used to select the 423 participants. Firstly, the sample was stratified based on their department. Then the selection was proportionally allocated in each stratum depending on the numbers of healthcare providers in each stratum or department to assess their knowledge, attitude, and associated factors related to patients' confidentiality. After allocating samples in each stratum proportionally, a computer-generated simple random sampling technique was employed to select the study subjects in each department (Fig. 1).

### Study variables

The primary outcome variable of this study was knowledge and attitude towards patient confidentiality. The questionnaires used in this study were developed based on a review of related literature [20, 21]. Socio-demographic and work-related characteristics were used as independent variables in this study.

### Operational definitions

*Knowledge about patients' confidentiality* was assessed using seven items with “yes” and “no” responses. Each correct answer was equal to one point, while each incorrect answer was equal to zero points, with a height possible score of 7 for the knowledge part. A mean of 7 questions regarding Knowledge towards patient confidentiality was calculated. And those above the mean score were categorized as ‘good’ knowledge, and those below were categorized as ‘poor’ knowledge [20].

*Attitudes toward patient confidentiality* were assessed by using 14 questions with a 5 point Likert scale from ‘strongly disagree’ (score 1) to ‘strongly agree’ (score 5) [20]. The final score in the attitude section ranges from 14 to 70. A mean of the 14 questions of attitude towards patient’s confidentiality was calculated. Those above the mean value were categorized as ‘favorable’ attitude, and those below the mean value were categorized as ‘unfavorable’ attitude [20].

### Data collection tool and quality control

A self-administered, organized, and pre-tested questionnaire was created in English. The data collection process included two supervisors and ten data collectors. One-day training was given to the data collectors to eliminate ambiguities. A pre-test was conducted outside of the study area, in Gondar town health centers, with 10% of the study population. The validity and reliability of the data collection instrument were assessed using the pre-test results. The Cronbach alpha value for the attitude questions was 0.82, whereas the Cronbach alpha value for the knowledge questions was 0.76. These figures show that the questionnaire is highly reliable.

### Data processing and analysis

The data entry was performed using Epi Data version 4.6 software packages and analyzed using Statistical Package for Social Sciences (SPSS) version 25. Descriptive statistics were computed to describe the socio-demographic variables and health professionals’ knowledge and attitudes about patient confidentiality and data sharing. Bi-variable and multivariable binary logistic regression analyses were done to measure the association between the dependent and independent variables. In the bi-variable regression analysis, variables with a *p* value of less than 0.2 were included in the multivariable regression analysis to assess their adjusted impacts on the dependent variables. Odds ratio with 95% confidence level and *P* value were calculated to ascertain the strength of association and to decide statistical significance. For all significantly associated variables, the cut-off value was *p* < 0.05. Before conducting the logistic regression model, assumptions of multi-collinearity were checked. The result revealed all the variance inflation factor (VIF) values less than three, which confirmed the absence of multi-collinearity.

## Result

### Description of participant’s socio-demographic and work-related characteristics

Of 423 participants, 410 responded to a questionnaire with a 96.9% response rate. The mean age of the participants was 28.12(SD ± 5.16) years which ranges from 21 to 50 years. The majority 271(66.1%) of the study participants were male and most of them 334 (81.5%) were orthodox religious followers. In terms of the educational level of the health professional, more than half 228 (55.6%) of participants have a BSc degree (Table 1). Of the total respondents, above three fourth 79.8% health professionals had below five years of work experience. A majority 47.8% of respondents were nurse professionals. Almost all 95.4% health professionals had direct contact with the patients and around 39% had visits above 40 patients per day. The results showed that about 5.9% of health professionals faced more than two ethical dilemmas daily while treating patients. In addition, 44.1% of the participants were taking training on medical ethics (Table 1).

**Table 1:** Socio-demographic and work-related characteristics of study participants (N = 410)

Socio-demographic characteristics	Frequency	Percentage
Sex		
Male	271	66.1
Female	139	33.9
Educational level		
Diploma	126	30.7
BSc degree	219	53.4
MD. doctor	28	6.8
MSc and specialist	37	9.0
Age		
≤ 24 years	101	24.6
25–34 years	254	62.0
≥ 35 years	55	13.4
Religion		
Orthodox	334	81.5
Muslim	58	14.1
Protestant	15	3.7
Others^a^	3	0.7
Marital status		
Single	274	66.8
Marred	136	33.2
Income in Ethiopian Birr		
> 6000	306	74.6
3000–6000	75	18.3
< 3000	29	7.1
Work experience		
> 5 years	126	30.7
≤ 5 years	284	69.3
Department		
Physician	39	9.5
Nurse	196	47.8
Pharmacy	32	7.8
Laboratory	39	9.5
Midwifery	60	14.6
Others^b^	44	10.7
Direct contact with the patients		
Yes	385	93.9
No	25	6.1
Numbers of the patient served per day		
> 40	165	40.2
30–40	114	27.8
< 30	131	32.0
Numbers of ethical dilemmas faced daily		
> 2	24	5.9
≤ 2	386	94.1
Training on medical ethics		
Yes	190	46.3
No	220	53.7

^a^Catholic, adventist

^b^Psychiatry, physiotherapy, anesthesia, optometry, environmental health

### Health professionals’ knowledge about patients’ confidentiality

**Fig. 2:**
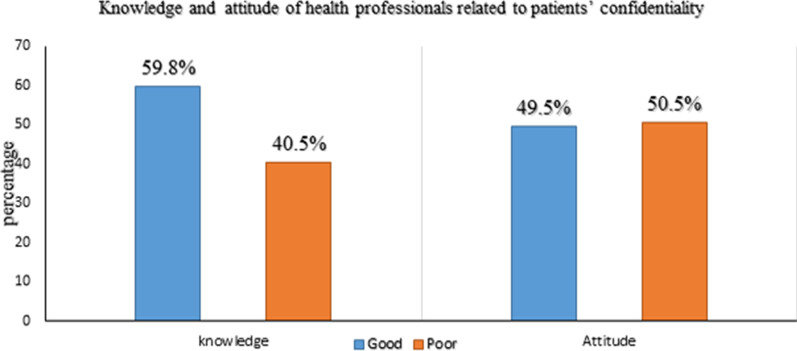
Health professional knowledge and attitude related to patients’ confidentiality

Of the total participants, 59.8% with [95% CI (54.9–64.5%)] had good knowledge about confidentiality with a mean score of 3.91(SD ± 1.39) (out of a maximum of 7 points) (Fig. 2). From the knowledge questionnaire, most of the respondents 358(87.3%) were said ‘access to medical records should be governed by law’ and 183(44.6%) argued that non-medical information is also confidential. Furthermore, 291(71%) health professionals were aware that third-party insurance companies did not access patient examination results (such as insurance companies) without patient consent. However, only 115(28.0%) of participants knew that policies were not allowed to access medical records freely (Table 2).

**Table 2:** Health professionals’ responses related to knowledge about confidentiality (N = 410)

Knowledge about patients’ confidentiality	No	Yes
	N (%)	N (%)
Does confidentiality is governed by low	52 (12.7)	358 (87.3)
Does non-medical information confidential	227 (55.4)	183 (44.6)
Are policies allowed to access medical records freely	115 (28.0)	295 (72.0)
Can the third party access a result without patient consent	291 (71.0)	119 (29.0)
Can confidentiality be breached if a patient has died	203 (49.5)	207 (50.5)
Can patient confidentiality be breached if the disease contagious	111 (27.1)	299 (72.9)
Patient confidentiality can be breached if the disease is not contagious	265 (64.6)	145 (35.4)

### Health professionals attitude towards patients’ confidentiality

Of the total participants, 49.5% with [95% CI (44.6–54.3%)] had a favorable attitude towards confidentiality with a mean score of 42.8(SD ± 8.90) (out of a maximum of 70 points) (Fig. 2). Table 3 illustrates that about 126(30.7%) of participants agreed that confidentiality affects the patient in any way, and 299(72.9%) believed they don’t allow non-medical personnel to enter the examination room while they are discussing with patients. Of all respondents, 220(53.7%) and 162(39.5%) participants use lock systems and computers to store patient information.

**Table 3:** Health professionals’ responses related to attitudes towards patient confidentiality (N = 410)

Attitude towards patients’ confidentiality	SD (%)	DA (%)	N (%)	A (%)	SA (%)
Confidentiality does not affect patient in any way	51(12.4)	75(18.3)	8(2.0)	182(44.4)	92(22.9)
I discuss the patient condition with them in front of others	34(8.3)	86(21.0)	58(14.1)	181(44.1)	51(12.4)
While I’m with patients I allow non-medical personnel to enter the examination room	112(27.3)	187(45.6)	15(3.7)	67(16.3)	29(7.1)
I use Lock to store patient information	16(3.9)	94(22.9)	80(19.5)	131(32.0)	89(21.7)
I use personal computer to store patient information	33(8.0)	118(28.8)	97(23.7)	118(28.8)	44(10.7)
I send patient information online	44(10.7)	116(28.3)	163(39.8)	67(16.3)	20(4.9)
I send patient information with phone	40(9.8)	109(26.6)	166(40.5)	72(17.6)	23(5.6)
I deal with the information of patients with sensitive diseases with more cautions	11(2.7)	85(20.7)	26(6.3)	181(44.1)	107(26.1)
I use virus protection software on my devices	23(5.6)	79(19.3)	97(23.7)	131(32.0)	80(19.5)
I discussed patient condition with my colleagues	20(4.9)	56(13.7)	65(15.9)	232(56.6)	37(9.0)
I discussed patient condition with colleagues in open space	52(12.7)	99(22.7)	135(32.9)	111(27.1)	19(4.6)
I discussed the patient condition with my friend outside the workplace	78(19.0)	123(30.0)	122(29.8)	72(17.6)	15(3.7)
I don’t leave my patient information on the desk	66(16.1)	153(37.3)	26(6.3)	126(30.7)	39(9.5)
I make or receive the phone call about my patient condition near others	64(15.6)	157(38.3)	32(7.8)	117(28.5)	40(9.8)

SD, strongly disagree; DA, disagree; N, neutral; A, agree; SA, strongly agree

### Factors associated with health professionals’ knowledge about patients’ confidentiality

Bi-variable and multivariable binary logistic regression analyses were done to measure the association between Health professionals’ knowledge towards patients’ confidentiality and independent variables. In bi-variable regression, Sex of participants, Age of the respondents, Work experience, Training on medical ethics, Numbers of the patient served, Direct contact with the patients, Numbers of ethical dilemmas faced, Income of participants were the candidates’ variables for health professionals’ knowledge towards confidentiality for the multivariable regression analysis (*P* < 0.2). With the multivariable regression model sex of respondents, training on medical ethics, number of ethical dilemmas faced were significantly associated factors for health professional knowledge towards patients’ confidentiality (Table 4). This means that being male was (AOR = 1.63, 95% CI [1.03–2.59]) times more likely to have good knowledge towards patient confidentiality as compared to females after controlling for other factors. Health professionals taking training on medical ethics were (AOR = 1.73, 95% CI = [1.11–2.70]) times more likely to have a good knowledge towards patients’ confidentiality as compared to their counterparts. Similarly, health professionals who faced more ethical dilemmas were (AOR = 3.07, 95% CI [1.07–8.79]) times more likely to have good knowledge than those who faced fewer ethical dilemmas.

**Table 4:** Factors associated with health professionals’ knowledge about patients’ confidentiality (N = 410)

Characteristics	Knowledge		COR (CI 95%)	AOR (CI 95%)	*P* value
	Good (%)	Poor (%)			
Sex					
Male	172(42.0)	99(24.1)	1.57(1.03–2.37)	1.63(1.03–2.59)	0.035*
Female	73(17.8)	66(16.1)	1	1	
Age					
≤ 24 Years	67(16.3)	34(8.3)	2.04(1.04–3.99)	1.87(0.86–4.04)	0.112
25–34 Years	151(36.8)	103(25.1)	1.52(0.84–2.72)	1.34(0.71–2.53)	0.361
≥ 35 Years	27(6.6)	28(6.8)	1	1	
Work experience					
> 5 years	72(17.6)	54(13.2)	0.85(0.55–1.31)	0.86(0.53–1.41)	0.566
≤ 5 years	173(42.2)	111(27.1)	1	1	
Training on medical ethics					
Yes	126(30.7)	64(15.6)	1.67(1.11–2.49)	1.73(1.11–2.70)	0.015*
No	119(29.0)	101(24.6)	1	1	
Direct contact with the patients					
Yes	233(56.8)	152(37.1)	1.66(0.73–3.73)	1.69(0.71–4.01)	0.227
No	12(2.9)	13(3.2)	1	1	
Numbers of patient served per day					
> 40 patients	96(23.4)	69(16.8)	0.94(0.59–1.50)	0.98(0.58–1.66)	0.966
30–40 patients	71(17.3)	43(10.5)	1.12(0.67–1.87)	1.03(0.60–1.77)	0.900
< 30 patient	78(19.0)	53(12.9)	1	1	
Numbers of ethical dilemma faced daily					
> 2 Times	19(4.6)	5(1.2)	2.69(0.98–7.35)	3.07(1.07–8.79)	0.036*
≤ 2 Times	226(55.1)	160(39.0)	1	1	
Income (Ethiopian birr)					
> 6000	176(42.9)	130(31.7)	0.35(0.14–0.89)	0.40(0.15–1.07)	0.068
3000–6000	46(11.2)	29(7.1)	0.41(0.15–1.13)	0.40(0.14–1.17)	0.096
< 3000	23(5.6)	6(1.5)	1	1	

*Significance at *p* < 0.05

### Factors associated with health professionals’ attitude towards patients’ confidentiality

In both bi-variable and multivariable analysis training on medical ethics, direct contact with the patients, Numbers of patient visits, and numbers of ethical dilemmas faced were significant variables to the attitude of health professionals towards patient confidentiality (Table 5).

**Table 5:** Factors associated with health professionals’ attitude towards patients’ confidentiality (N = 410)

Characteristics	Attitude		COR (CI 95%)	AOR (CI 95%)	*P* value
	Favorable (%)	Unfavorable (%)			
Sex					
Male	144(35.1)	127(31.0)	1.53(1.01–2.32)	1.04(0.65–1.67)	0.844
Female	59(14.4)	80(19.5)	1	1	
Age					
≤ 24 Years	56(13.7)	45(11.0)	1.29(0.66–2.49)	1.64(0.73–3.67)	0.228
25–34 Years	120(29.3)	134(32.7)	0.92(0.51–1.66)	1.02(0.51–2.04)	0.939
≥ 35 Years	27(6.6)	28(6.8)	1	1	
Work experience					
> 5 years	71(17.3)	55(13.4)	1.48(0.97–2.26)	1.40(0.84–2.32)	0.191
≤ 5 years	132(32.2)	152(37.1)	1	1	
Educational level					
MSc and specialist	24(5.9)	13(3.2)	1.90(0.89–4.07)	2.17(0.82–5.74)	0.117
Medical doctor	15(3.7)	13(3.2)	1.19(0.52–2.70)	1.32(0.49–3.55)	0.575
BSc degree	102(24.9)	117(28.5)	0.90(0.58–1.39)	0.89(0.48–1.65)	0.721
Diploma	62(15.1)	64(15.6)	1	1	
Training on medical ethics					
Yes	105(25.6)	85(20.7)	1.53(1.04–2.27)	2.30(1.42–3.72)	0.001*
No	98(23.9)	122(29.8)	1	1	
Direct contact with the patients					
Yes	195(47.6)	190(46.3)	2.18(0.91–5.17)	3.06(1.12–8.34)	0.029*
No	8(2.0)	17(4.1)	1	1	
Numbers of patient served per day					
> 40 patients	99(24.1)	66(16.1)	2.77(1.72–4.45)	4.38(2.46–7.80)	0.000*
30–40 patients	58(14.1)	56(13.7)	1.91(1.14–3.19)	1.96(1.12–3.43)	0.017*
< 30 patient	46(11.2)	85(20.7)	1	1	
Numbers of ethical dilemma faced daily					
> 2 Times	19(4.6)	5(1.2)	4.17(1.52–11.39)	3.56(1.23–10.26)	0.019*
≤ 2 Times	184(44.9)	202(49.3)	1	1	
Income (Ethiopian birr)					
> 6000	147(35.9)	159(38.8)	0.48(0.21–1.08)	0.41(0.15–1.12)	0.083
3000–6000	37(9.0)	38(9.3)	0.51(0.21–1.24)	0.44(0.16–1.19)	0.110
< 3000	19(4.6)	10(2.4)	1	1	
Knowledge					
Good	132(32.2)	113(27.6)	1.54(1.03–2.30)	1.36(0.88–2.11)	0.166
Poor	71(17.3)	94(22.9)	1	1	

*Significance at *p* < 0.05

Health professionals taking on medical ethics were (AOR = 2.30, 95% CI [1.42–3.72]) times more likely to have a favorable attitude towards patient confidentiality when compared to those who didn’t take any pieces of training on medical ethics. Health professionals who had direct contact with the patients were (AOR = 3.06, 95% CI [1.12–8.34]) times more likely to have a favorable attitude towards patient confidentiality than those who didn’t have direct contact with the patients. Health professionals who visited more patients daily (more than 40 and 30–40) were approximately (AOR = 4.38, 95% CI [2.46–7.80]) and (AOR = 1.96, 95% CI [1.12–3.43]) times more likely to have a favorable attitude towards patients’ confidentiality when compared to those who visited less than 30 patients daily. Additionally, respondents who faced more ethical dilemmas were (AOR = 3.56, 95% CI [1.23–10.26]) times more likely to have a favorable attitude towards patients’ confidentiality than those who faced fewer ethical dilemmas.

## Discussion

This study examines health professionals’ knowledge and attitude towards patient confidentiality and associated factors in Northwest Ethiopia.

This study revealed that around 59.8% of respondents had good knowledge related to patient confidentiality. The finding is in line with two studies conducted in Iran 56.6% [22], 63% [23]. However, the results of this study demonstrated that health professionals’ good knowledge towards patient confidentiality was lower than studies conducted in Spain 68% [24] and Tehran university medical school 65% [25]. The difference could be that health professionals working in high-resource countries are more informed about patients' privacy in their daily lives and recognize the relative benefit of patient confidentiality [26]. The other reasons for the disparity could be explained by the fact that approximately 75% of participants had less than 5 years of professional experience in the current study, and they were also considerably younger than in the Spanish study [24]. Furthermore, the participants in Spain were all physicians, who are supposed to have better clinical data management and specific training [24].

In this study, 49.5% of participants had a favorable attitude towards patient confidentiality. This finding is supported by the study conducted in northern Jordan 52.4% [20]. However, this finding is lower than the study conducted in Turkey (64.4%) physicians strongly agreed to protect patient confidentiality [27]. The possible reason could be that difference awareness among health professionals in different countries results in a good level of attitude.

The study also found factors associated with health professionals’ knowledge and attitude regarding patient confidentiality. The sex of respondents, training on medical ethics, and the number of ethical dilemmas faced was all significantly associated factors of health professional knowledge towards patients’ confidentiality. Likewise, training on medical ethics, direct contact with the patients, Numbers of patient visits, and numbers of ethical dilemmas faced were significant variables to the attitude of health professionals towards patient confidentiality.

Among the factors associated with knowledge, being males were more likely to have good knowledge towards patient confidentiality than females. This finding is consistent with study findings from Jordan [20, 21], Spain [24], and the United States [28]. This might be due to males were more access to information and technology and there is high information sharing between them [21]. Furthermore, the number of ethical dilemmas experienced and training on medical ethics were revealed to be predictive variables for both knowledge and attitude. Health professionals taking training on medical ethics were more likely to have a good knowledge and attitude related to patients’ confidentiality than those who had not taken the training. The greatest strategy to ensure adherence to confidentiality laws was to provide training on medical ethics, where health organizations would routinely update all health professionals on guidelines and strategies to prevent sensitive information disclosure [21, 29, 30]. Furthermore, the legislature's role is critical, not just in terms of legal norms to safeguard patient confidentiality, but also in terms of punishments when inappropriate behavior occurs [31]. And this finding is supported by a study conducted in Barbados [32], Vietnam [33]. Besides this, this study also found that health professionals who faced more ethical dilemmas were more likely to have good knowledge and attitude as compared to those who faced a less ethical dilemma. According to Hariharan et al. suggestions, health professionals may not report such problems to their seniors and try to solve them [32]. This may be the possible reasons for facing more ethical dilemma and trying to solve by themselves to have positive knowledge and attitude towards patient confidentiality.

In addition, direct contact with the patients and the number of patient visits were associated with a favorable attitude towards patient confidentiality. Respondents who have direct contact with the patients were more aware of confidentiality. This could be because the health of practitioners that deal with patients regularly are more familiar with confidentiality rules and strategies[22]. Besides this, health professionals who visit more patients per day were more likely to have a favorable attitude related to patient confidentiality. This might be because when health professionals serve more patients per day, they get a lot of challenges which helps to change their attitude to maintain the patient's information confidentially.

## Conclusion

The findings of this study revealed that health professionals have a limited attitude towards patient confidentiality but have relatively good knowledge. The sex of respondents, training on medical ethics, and many ethical dilemmas faced were significantly associated factors of health professional knowledge towards patients’ confidentiality. Likewise, training on medical ethics, direct contact with the patients, Numbers of patient visits, and numbers of ethical dilemmas faced were significant variables to the attitude of health professionals towards patient confidentiality. Providing a continuing medical ethics training package for health workers before joining the hospital and in between the working time could be recommended to improve health professionals' knowledge and attitude towards patient confidentiality.

### Strength and limitations

The findings from this study provide valuable information on health professionals' knowledge and attitude related to patients' confidentiality in resources limited countries. There are some limitations to this study. First, this study was an institution-based cross-sectional survey; only health professionals who came during the data collection period were interviewed.

## Supplementary Information

**Additional file 1:** Supplementary SPSS data on Knowledge of Patient Confidentiality.

**Additional file 2:** Supplementary SPSS data on attitude towards Patient Confidentiality.

## Data Availability

All the data generated or analyzed during this study are included in this published article and supplementary information [SPSS Data Knowledge and SPSS Data Attitude].

## Abbreviations

AOR: Adjusted odds ratio
CI: Confidence interval
Epi-info: Epidemiological information
ICT: Information communication technology
SPSS: Statistical package for social science
